# Smad2-Dependent Downregulation of miR-30 Is Required for TGF-β-Induced Apoptosis in Podocytes

**DOI:** 10.1371/journal.pone.0075572

**Published:** 2013-09-26

**Authors:** Shaolin Shi, Liping Yu, Taoran Zhang, Haiying Qi, Sandhya Xavier, Wenjun Ju, Erwin Bottinger

**Affiliations:** Department of Medicine, Mount Sinai School of Medicine, New York, New York, United States of America; Fondazione IRCCS Ospedale Maggiore Policlinico & Fondazione D’Amico per la Ricerca sulle Malattie Renali, Italy

## Abstract

Transforming growth factors beta (TGF-β) are multi-functional cytokines capable of inducing apoptosis in epithelial cells, including glomerular podocytes. We and others have previously shown that podocyte-selective genetic deletion of the microRNA (miR)-processing enzyme, Dicer, caused glomerulosclerosis that was associated with podocyte apoptosis, and the miR-30 family was implicated in the process. Here, we report that apoptosis-associated genes were highly enriched among the predicted targets of miR-30 when compared with randomly selected miRs (26% vs. 4.5 ± 2.1%) or with the known TGF-β-regulated miR-192 (6%), miR-216a (5.1%), and miR-217 (0%). miR-30 family members were abundantly expressed in podocytes in normal mice but were downregulated in albumin/TGF-β transgenic mice with podocyte apoptosis and glomerulosclerosis. *In vitro*, TGF-β downregulated miR-30s in wildtype and Smad3-deficient, but not Smad2- or Smad2/Smad3-deficient, podocytes. The TGF-β-induced activation of caspase 3 and an increase in TUNEL-positive nuclei were significantly inhibited by the lentivirus-mediated overexpression of miR-30d, but not by a scrambled control miR, in podocytes. TGF-β stimulated the phosphorylation of pro-apoptotic p53 in podocytes with lentiviral expression of a scrambled miR, but not in podocytes expressing miR-30d. In contrast, miR-30d had no effect on the phosphorylation of pro-apoptotic p38 MAP kinase induced by TGF-β. Thus, we report that Smad2-dependent inhibition of miR-30s in podocytes is required for the activation of p53 and the induction of apoptosis by TGF-β. These results demonstrate a novel functional role for miR-30 in podocyte survival and indicate that the loss of miR-30 survival signaling is a novel and specific mechanism of TGF-β-induced podocyte apoptosis during glomerulosclerosis. We propose the therapeutic replacement of miR-30 as a novel strategy to prevent the podocyte apoptosis that is characteristic of progressive glomerular diseases.

## Introduction

Transforming growth factors beta (TGF-β) are pluripotent cytokines that have essential roles in development, homeostasis and major diseases [1], including most forms of progressive kidney disease [2]. Chronic progressive kidney diseases are uniformly characterized by loss of glomerular podocytes in part due to apoptotic cell death, resulting in glomerular capillary collapse, glomerulosclerosis, proteinuria, and loss of glomerular filtration [3,4].

Upon ligand stimulation, TGF-β receptor complexes expressed on podocytes activate multiple intracellular signaling pathways, including Smad-dependent, MAP kinase, and PI3K/Akt pathways, that determine podocyte cell survival or death [5,6]. For example, Smad3 is required for the activation of pro-apoptotic caspase 3, whereas Cd2ap-dependent activation of PI3K/Akt mediates cell survival signaling [6]. The relative activation of pro-apoptotic and pro-survival signaling may be TGF-β concentration (dose)-dependent where moderate concentrations promote cell cycle arrest and differentiation and increasing concentrations promote apoptosis associated with p38 MAP kinase activation [7]. *In vivo*, the transgenic overexpression of TGF-β, resulting in elevated circulating TGF-β levels, is sufficient to cause the podocyte apoptosis that characterizes the early manifestation of progressive glomerulosclerosis [8].

miRNAs are a group of small non-coding RNAs, each of which is capable of regulating the expression of hundreds of target genes simultaneously by interacting with target sequences in their 3’-untranslated regions, resulting in transcript degradation or repression of translation [9]. Cre/Lox-mediated deletion of Dicer, an RNase III enzyme required for the biogenesis of mature miRNAs, in selected renal cell types has demonstrated that miRNAs are critical for the homeostasis of renin-producing juxtaglomerular cells [10] and for the resistance of tubular epithelial cells to ischemia-reperfusion induced injury [11]. In addition, miR-192 promotes collagen production in glomerular mesangial cells in experimental diabetic nephropathy by targeting E-box repressors that control collagen expression [12]. miR-192 also mediates TGF-β/Smad3-induced tubulointerstitial fibrosis [13]. In experimental diabetic nephropathy models, TGF-β and miR-192 upregulate miR-216 and 217, which in turn inhibit their target molecule, Pten, resulting in Akt activation, survival and hypertrophy of glomerular mesangial cells [14]. Finally, miR-93 has been shown to modulate glomerular injury through vascular endothelial growth factor (VEGF) in diabetic nephropathy [15].

We and others have reported that the Cre/loxP-mediated deletion of Dicer selectively in glomerular podocytes of mice (Dicer^fl/fl^:NPSH2-Cre) results in progressive proteinuria and glomerulosclerosis that is associated with progressive podocyte apoptosis and depletion [16-18]. Among the 190 genes that are upregulated in the glomeruli of Dicer^fl/fl^:NPSH2-Cre mice, the predicted miR-30 targets were highly enriched, suggesting a role for miR-30 in the gene expression and homeostasis of podocytes [16].

The miR-30 family consists of 5 evolutionarily conserved members, miR-30a through -30e. Although the precise physiological roles of miR-30s remain poorly understood, miR-30 members may promote tumor invasion and metastasis by targeting Galphai2 in liver cancer cells [19]. miR-30s have been shown to inhibit apoptosis of cardiomyocytes via targeting p53 [20], whereas in breast tumor-initiating cells, they promote apoptosis and disrupt cell self-renewal [21]. In addition, miR-30 has been implicated in the epithelial-mesenchymal transition (EMT) or mesenchymal-epithelial transition (MET) via TGF-β signaling in anaplastic thyroid carcinomas [22]. Finally, miR-30s are involved in hepatobiliary [23] and pronephros [24] development.

In the current study, we report that miR-30s are expressed selectively and abundantly in glomerular podocytes in mice and that TGF-β profoundly downregulates miR-30 members in podocytes both *in vivo* and *in vitro*. Mechanistic studies demonstrated a novel and selective functional role for Smad2-dependent downregulation of miR-30 in the TGF-β-mediated activation of pro-apoptotic p53, and this pathway was required for TGF-β-induced podocyte apoptosis. Thus, we have identified a specific and novel cell survival mechanism in glomerular podocytes in which miR-30s prevent the activation of pro-apoptotic p53. Maintenance of sufficient miR-30 levels may provide a new therapeutic strategy to promote podocyte survival and prevent podocyte depletion in progressive glomerular diseases.

## Methods

### Ethics Statement

All animal studies were performed according to protocols approved by the Institutional Animal Care and Utilization Committee (IACUC No. 04-0386-00001-07) of Mount Sinai School of Medicine and adhered to NIH guidelines for animal care.

### Mouse and Cell lines

The albumin enhancer/promoter-driven porcine TGF-β (Alb/TGF-β) transgenic mice were obtained from Dr. Snorri Thorgeirsson [25]; Smad3 knockout mice were from Dr. Chuxia Deng [26]; a Smad2 conditional allele (loxP floxed) was generated as described [27]. To generate mice lacking both Smad2 and Smad3 in podocytes, Smad3 null, loxP floxed Smad2, and NPHS2-Cre transgenic mice were inter-crossed to obtain mice with a genotype of Smad3^-/-^/Smad2 ^fl/fl^/NPHS2-Cre. To establish conditionally immortalized podocyte lines from these mice, they were further crossed to Immortomouse® transgenic mice (Charles River, Wilmington, MA). Podocyte cell lines were generated following the previously described method [28]. The human podocyte line has been described previously [29].

### Lentiviral system

We purchased miR-30d- and scrambled control miR-expressing lentiviruses from GeneCopoeia (Rockville, MD) and followed the company’s instructions for preparation of lentiviral stocks.

### *In silico* predictions of miR-30 targets and function

To obtain a list of the most reliable miR-30 target genes, we retrieved the predicted targets that are evolutionarily conserved in mammals (including human, dog, mouse and rat) from three independent databases, TargetScan (http://www.targetscan.org/), PicTar (http://pictar.mdc-berlin.de) [30], and miRbase (http://www.ebi.ac.uk/enright-srv/microcosm/htdocs/targets/), and then selected the common genes as our predicted miR-30 targets. We used this target list for *in silico* analysis with Ingenuity Pathway Analyses (www.ingenuity.com/).

### Cell culture and lentiviral infection

Conditionally immortalized mouse podocytes were maintained in RPMI-1640 media containing 10% FBS under either permissive conditions (33 °C with 10 units/ml IFN-γ) or non-permissive conditions (37 °C without IFN-γ). Human podocytes were maintained as described [29]. To infect podocytes with lentivirus, the lentiviral stock was mixed with polybrene (1 µg/ml final concentration), and the solution was added to the podocytes after removing the culture medium. After 24 hours, the lentiviral mixture was replaced with fresh normal media containing 300 µg/ml G418. After 4-5 days, the podocytes were replated for TGF-β treatment or other assays.

### Quantitative real-time PCR of miRNAs

Small RNA or total RNA of podocytes was extracted after treatment using the *mirVana* miRNA extraction kit (Ambion, TX). miR-30 quantification in the RNA samples was conducted by qRT-PCR using the Ncode miRNA Amplification System (Invitrogen, Carlsbad, CA). An Applied Biosystems (Bedford, MA) 9700 PCR system was used for the qPCR. U6 RNA or 5S rRNA real-time PCR was performed simultaneously, and the Ct values were used to normalize the expression of miR-30s.

### Luciferase reporter assay

3’-UTR fragments of the predicted target genes of interest were amplified by PCR and inserted downstream of the pGL3 promoter (Promega, Madison, WI) via *HindIII*. The resultant constructs were co-transfected with a renilla luciferase normalization control into HEK293 cells using LipofectAmine 2000. After 24 hr, cell lysates were prepared and subjected to luciferase assays using the Dual-luciferase Report Assay System (Promega, Madison, WI).

### Western blotting

Cell lysates were made using RIPA buffer containing a protease inhibitor cocktail (Roche) and a Halt phosphatase inhibitor (Thermo Scientific). After 10% SDS-PAGE gel fractionation and membrane transfer, the blots were incubated with primary antibodies against cleaved caspase 3, phosphorylated Smad2, Smad2/Smad3, phosphorylated p38, total and phosphorylated p53, GAPDH or tubulin (all antibodies were purchased from Cell Signaling Technology, Beverly, MA).

### TUNEL assay

Rates of apoptosis in podocytes treated with TGF-β were analyzed by TUNEL assays using the ApopTag Red kit (Chemicon, Billerica, MA).

### miRNA *in situ* hybridization of kidney sections

miRNA locked nucleic acid (LNA) probes were purchased from Exiqon (Denmark). Mice were perfused with 10% formaldehyde, and kidneys were excised and incubated with 18% sucrose in PBS overnight at 4 °C. Kidney sections (15 µm) were cut and hybridized with LNA miRNA probes labeled with digoxigenin at 55 °C overnight. After washing, kidney sections were incubated with an anti-Dig antibody conjugated with alkaline phosphatase at room temperature for 3 hr followed by color development with NBT/BCIP (Roche) as substrates.

## Results

### miR-30s are abundant in podocytes and are downregulated by TGF-β *in vitro* and *in vivo*

**Figure 1:**
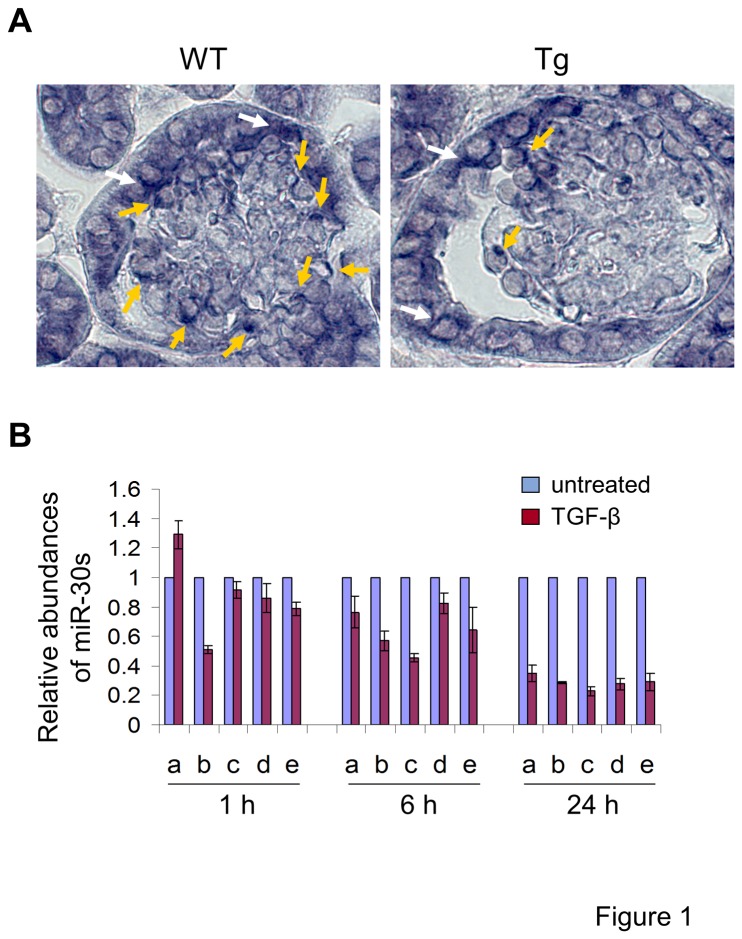
miR-30s are downregulated by TGF-β in glomerular podocytes *in vivo* and *in vitro*. **A**. miR-30d transcripts were abundantly detected by *in* *situ* hybridization in podocytes (yellow arrows) and parietal epithelial cells (white arrows) in adult wildtype (wt) control mice, but not in Alb-TGF-β transgenic (Tg) mice; **B**. miR-30a, -30b, -30c, -30d, and -30e were significantly downregulated in cultured human podocytes after 6 and 24 hr of TGF-β treatment (5 ng/ml). Bar graph shows the mean ± S.D. of the relative abundance of miR-30 members in podocytes untreated (white bars) and treated (black bars) with TGF-β for 1, 6, and 24 h.

Our previous bioinformatics analysis of the glomerular gene expression profiles of Dicer^fl/fl^:NPSH2-Cre mice revealed an enrichment of predicted miR-30 target genes among the upregulated genes [16], suggesting that miR-30s are expressed in podocytes/glomeruli and that their deficiencies due to Dicer deletion contributed to the gene expression changes observed in the podocytes/glomeruli of the mice. As Alb-TGF-β transgenic mice [8] phenocopy the progressive glomerulosclerosis associated with podocyte apoptosis and depletion observed in Dicer^fl/fl^:NPSH2-Cre mice [16-18], we speculated that the putative expression of miR-30s in podocytes might be downregulated in these mice. Thus, we performed *in situ* hybridization studies on kidney sections using a miR-30d LNA probe. In adult control mice, miR-30d was abundantly present selectively in podocytes and parietal glomerular epithelial cells, but was absent in glomerular endothelial and mesangial cells (Figure 1A). In contrast, miR-30d expression was greatly reduced in podocytes of Alb-TGF-β mice (Figure 1A). Next we performed quantitative PCR analyses of miR-30a, -30c and -30d in the glomerular RNA from the mice, and the results indicated that these miR-30s were all downregulated in the glomeruli of Alb-TGF-β mice compared with controls (Figure S1). Moreover, we examined the precursors of these miR-30s in these RNA samples by qPCR, and the result showed that they were also downregulated in the glomeruli of Alb-TGF-β mice (Figure S2), suggesting that TGF-β regulates miR-30 expression at the transcription level. Finally, TGF-β treatment of human podocytes cultured under non-permissive or permissive conditions significantly reduced the levels of all five miR-30 family members beginning at 6 hrs, as determined by qRT-PCR (Figure 1B). Similar results were obtained using conditionally immortalized murine podocytes (data not shown). These findings demonstrate that miR-30s are abundantly expressed in the podocytes and parietal epithelial cells of glomeruli, and TGF-β downregulates miR-30 expression in podocytes both *in vivo* and *in vitro*.

### Apoptosis associated genes are highly enriched among the predicted miR-30 targets

**Figure 2:**
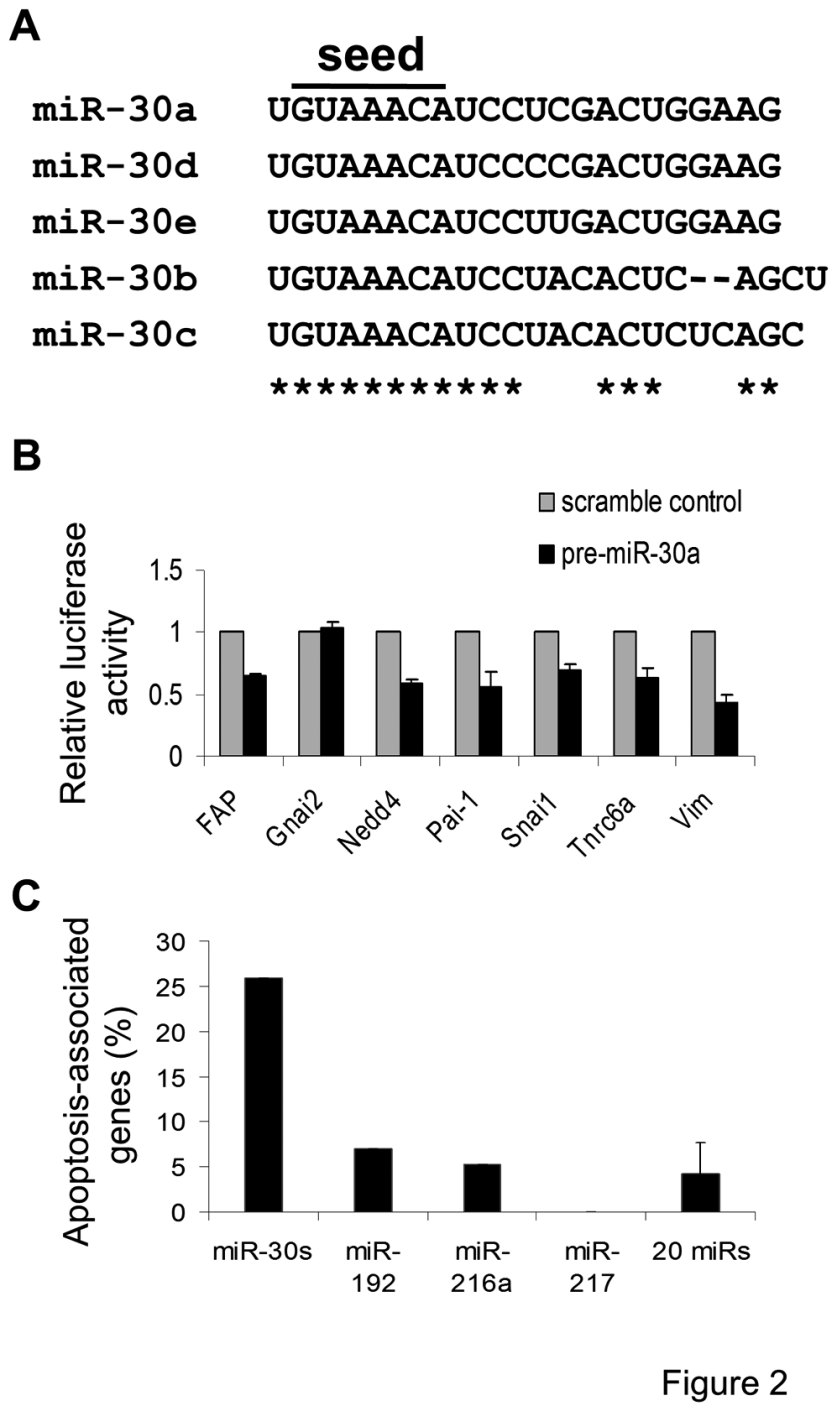
Genes associated with apoptosis are significantly enriched among the predicted target genes of miR-30s. **A**. Alignment of the sequences of mature miR-30 family members with seed sequence motifs (indicated by a line). **B**. Bar graph showing the mean ± S.D. of the activity of luciferase reporter constructs carrying 3’ UTR sequence fragments of seven genes randomly chosen from the 155 predicted miR-30 target genes. Reporter constructs were cotransfected with either a scrambled miR expression construct (control) or a synthetic miR-30 precursor (pre-miR-30a). The results were normalized by the activity of renilla luciferase, the expression construct of which was co-transfected. **C**. Bar graph shows the fraction of genes annotated with the biological process, ‘apoptosis’ (Ingenuity Systems), among all the predicted target genes of TGF-β-regulated miR-30s, miR-192, miR-216a, and miR-217 or the mean ± S.D. of 20 randomly chosen miRs that are not known to be regulated by TGF-β (control).

A typical miR is predicted to target hundreds of genes based on the presence of its recognition motif(s) in the 3’ untranslated regions (UTRs) of the genes. To determine whether a putative functional role of miR-30 could be predicted by *in silico* analysis of miR-30 target genes, we took a stringent approach and searched for potential miR-30 target genes that not only carry evolutionarily conserved miR-30 recognition motifs in their 3’-UTRs (Figure 2A) but also are consistently predicted by the three independent miR databases.

There were 873 genes predicted to be miR-30 targets by TargetScan, 634 by PicTar, and 1,566 by miRbase. Among these genes, 155 were predicted to be miR-30 targets in all three databases and were conserved in human, dog, rat and mouse (Table S1 in File S1).

To validate the *in silico* predictions experimentally, we generated luciferase reporter vectors containing 3’-UTRs with miR-30 recognition sequences from 7 of the predicted target genes. Co-transfection of the miR-30a precursor with these reporter constructs resulted in significantly reduced luciferase activity in 6 out of the 7 reporter vectors when compared with the scrambled control precursor (Figure 2B). Our results suggest that a high estimated percentage (~ 86%) of the 155 genes could be experimentally validated as genuine targets of miR-30.

Because TGF-β induces podocyte apoptosis and podocyte apoptosis is a mechanism for the podocyte depletion that leads to progressive glomerulosclerosis in both Dicer^fl/fl^:NPSH2-Cre and Alb/TGF-β transgenic mice, we determined the frequency of target genes that were associated with apoptosis in TGF-β-regulated and random control miRNAs. Functional annotations were available in the Ingenuity Pathway Analysis software for 116 of the 155 predicted miR-30 targets. Thirty out of 116 (26%) of the annotated miR-30 target genes were associated with apoptosis (Figure 2C, Table S2 in File S1). In contrast, only 4.5 ± 2.1% of the predicted target genes of 20 randomly selected human miRs used as controls were associated with apoptosis (Figure 2C). In addition, the frequencies of apoptosis-associated target genes of miR-192, miR-216a, and miR-217, all previously reported to be regulated by TGF-β in kidney disease [12-14,31], were not significantly different from random control miRNAs (Figure 2C). Together, these results suggest that among the various miRs regulated by TGF-β in kidney disease, the TGF-β-induced downregulation of miR-30 may regulate apoptosis-associated target genes and their associated apoptotic pathways.

### Lentiviral miR-30 expression sustains miR-30 levels in podocytes treated with TGF-β

**Figure 3:**
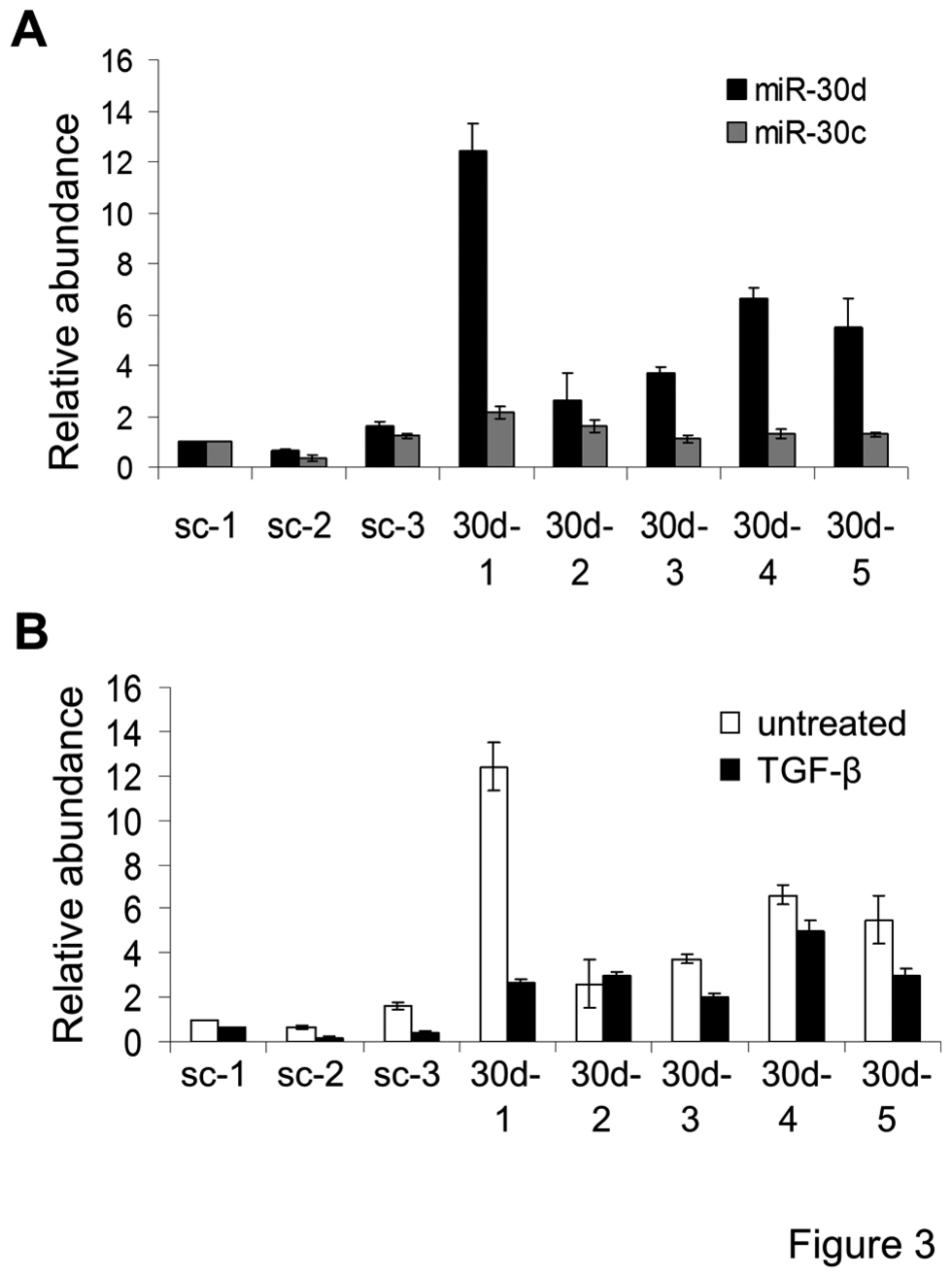
Lentiviral expression of miR-30d in infected podocytes sustains miR-30d levels upon TGF-β treatment. **A**. Bar graph showing the mean ± S.D. of the relative abundance of miR-30d (black bars) and miR-30c (gray bars) in podocyte clones stably expressing scrambled miR (sc-1, sc-2, sc-3) or miR-30d (30d-1, -2, -3, -4, -5). **B**. Bar graph showing the relative abundance of miR-30d levels in three control clones (sc-1, -2, and -3) and five miR-30d-expressing clones left untreated (white bars) or treated with TGF-β (5 ng/ml) (black bars) for 48 hr.

To investigate whether miR-30 downregulation by TGF-β had any role in podocyte apoptosis, one of the miR-30 family members, miR-30d, was studied as a representative member of the family. We used a lentiviral system to overexpress miR-30d to maintain miR-30d levels in podocytes during TGF-β treatment. Mouse podocytes were infected with a miR-30d-expressing lentivirus followed by G418 treatment to eliminate the uninfected cells. Five independent stable clones were examined for miR-30d expression by qRT-PCR, and all were found to have an increased level of miR-30d (Figure 3A). In response to TGF-β treatment, miR-30d was downregulated in the clones expressing scrambled control miRNA (Figure 3B). In contrast, miR-30d levels remained high in the miR-30d-overexpressing clones in the presence of TGF-β (Figure 3B). The small decrease of miR-30d by TGF-β in the lentiviral miR-30d-expressing clones might be caused by endogenous miR-30d downregulation by TGF-β. Together, these results demonstrated that miR-30d levels was sustained by lentiviral miR-30d expression in podocytes treated with TGF-β.

### Sustained expression of miR-30 inhibits TGF-β induced apoptosis of podocytes

**Figure 4:**
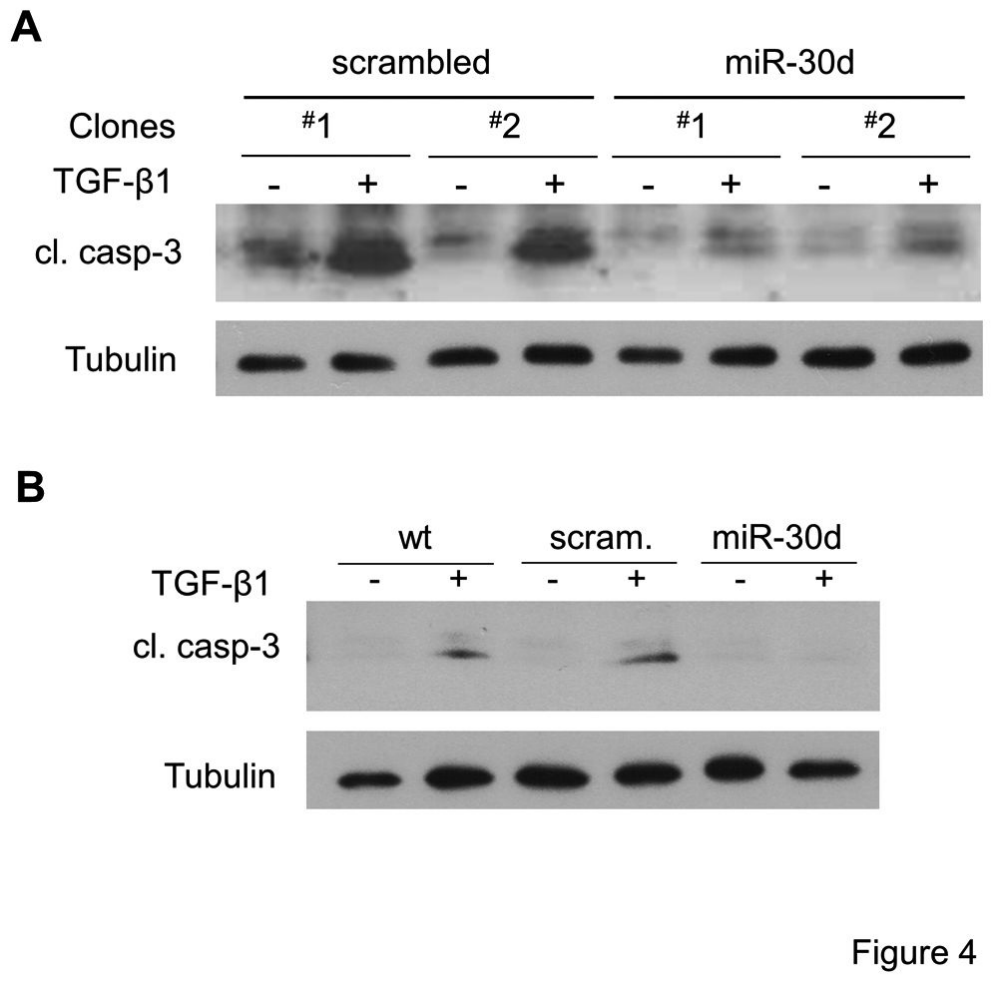
Lentiviral expression of miR-30d prevents TGF-β-induced caspase-3 activation. Immunoblots showing cleaved caspase-3 levels **A**. in two independent clones each of scrambled miR- or miR-30d-expressing podocytes left untreated (-) or treated with TGF-β (5 ng/ml) (+) for 48 hr; or **B**. in uninfected wildtype podocytes (wt) and pooled podocytes infected with scrambled miR (Scram)- or miR-30d-expressing lentivirus left untreated (-) or treated with TGF-β (+) (tubulin is shown as loading control).

Exogenous miR-30d-expressing podocyte clones and scrambled miR-expressing control clones were treated with TGF-β for 48 hrs. As expected, TGF-β induced the cleavage of caspase 3 in two of the control clones, but not in the miR-30d-expressing clones (Figure 4A). Similarly, TGF-β induced caspase 3 cleavage in wildtype podocytes and pooled scramble miR-expressing clones, but not in pools of miR-30d-expressing clones (Figure 4B).

**Figure 5:**
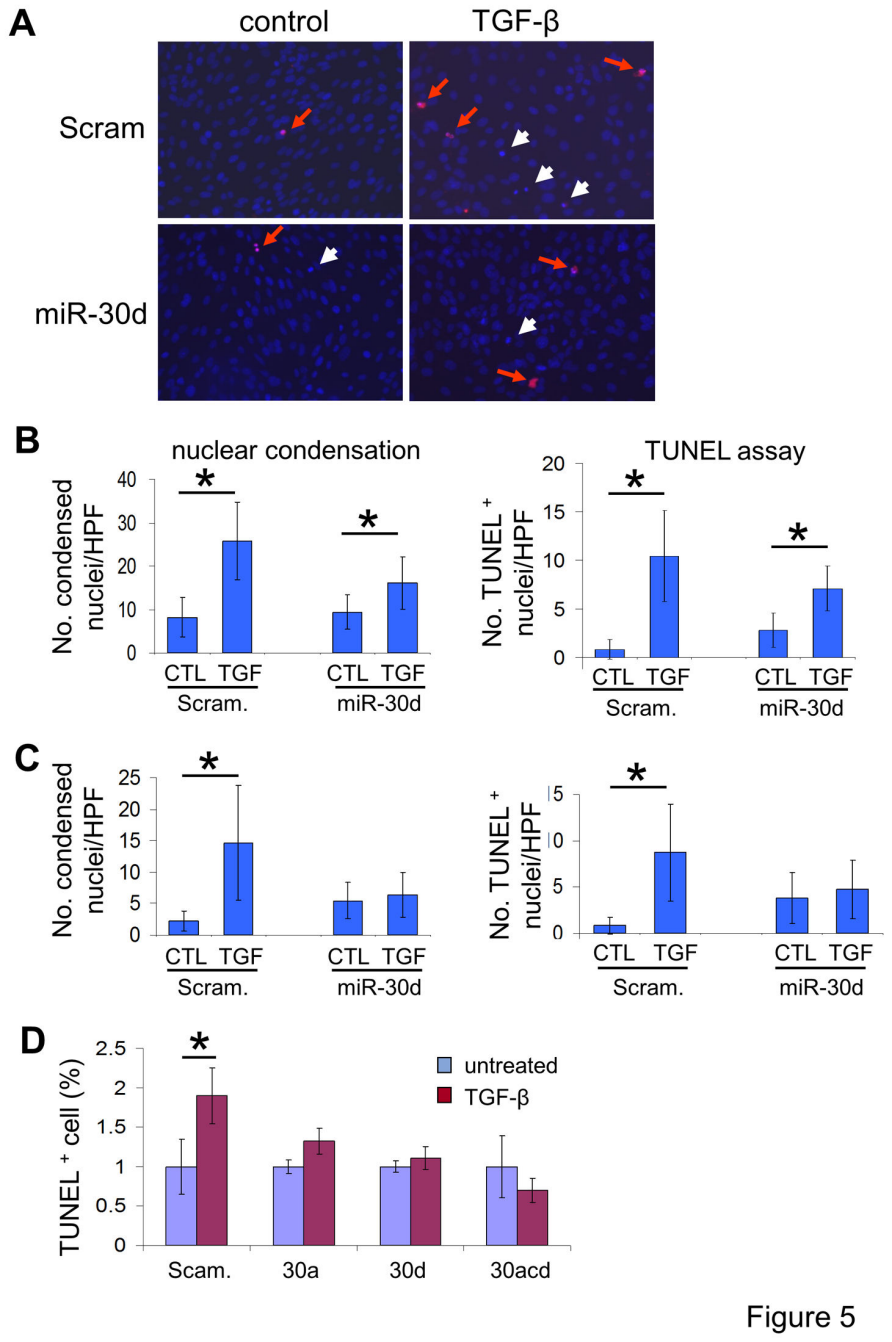
Lentiviral expression of miR-30s prevents TGF-β-induced apoptosis in podocytes. **A**. Representative images showing TUNEL-positive nuclei (red arrows) and condensed nuclei (white arrows) in conditionally immortalized murine podocytes expressing scrambled miR (Scram) or miR-30d left untreated or treated with TGF-β for 48 hr (20X). **B**. and C. Bar graphs showing the mean ± S.D. of the condensed nuclei or TUNEL-positive nuclei per high power field (HPF, 20X) in the experiments described in A. Fifty HPFs were examined for each sample (bar). B. Quantitation of apoptosis rates under permissive proliferating culture conditions (33 °C, + IFN-γ). C. Quantitation of apoptosis rates under non-permissive differentiating culture conditions (37 °C, - IFN-γ); D. Bar graph demonstrating the fraction of TUNEL-positive nuclei in human podocytes infected with lentiviral vectors to express either scrambled control miR (Scram) or miR-30a (30a), miR-30d (30d), or combined miR-30a, -30c, -30d (30acd) left untreated (blue bars) or treated with TGF-β (5 ng/ml) for 48 hr (red bars). * denotes p < 0.05.

Next, we quantitated both condensed and TUNEL-positive nuclei to evaluate podocyte apoptosis (Figure 5A). TGF-β treatment (5 ng/ml) for 48 hrs significantly increased the number of condensed nuclei and the number of TUNEL-positive nuclei in scrambled miR-expressing control podocytes, but these numbers were not highly increased in miR-30d-expressing podocytes cultured under either permissive (Figure 5B) or non-permissive conditions (Figure 5C). Similar results were obtained when these experiments were repeated using human podocytes (data not shown).

Finally, we repeated these experiments using lentiviral miR-30a or a combination of miR-30a, -30c, and -30d in comparison with a scrambled miR control and miR-30d (Figure 5D). TGF-β treatment significantly increased the rate of apoptosis in podocytes expressing scrambled miR (Figure 5D) and in human podocytes. In contrast, TGF-β had no effect on the apoptotic rates of podocytes with lentiviral expression of either miR-30a, miR-30d, or miR-30a/30c/30d combined (Figure 5D). These results indicate that the exogenous expression of miR-30s to sustain cellular level of miR-30s protects podocytes against TGF-β-induced apoptosis.

### miR-30 downregulation by TGF-β is mediated by Smad2-dependent signaling and does not require Smad3

**Figure 6:**
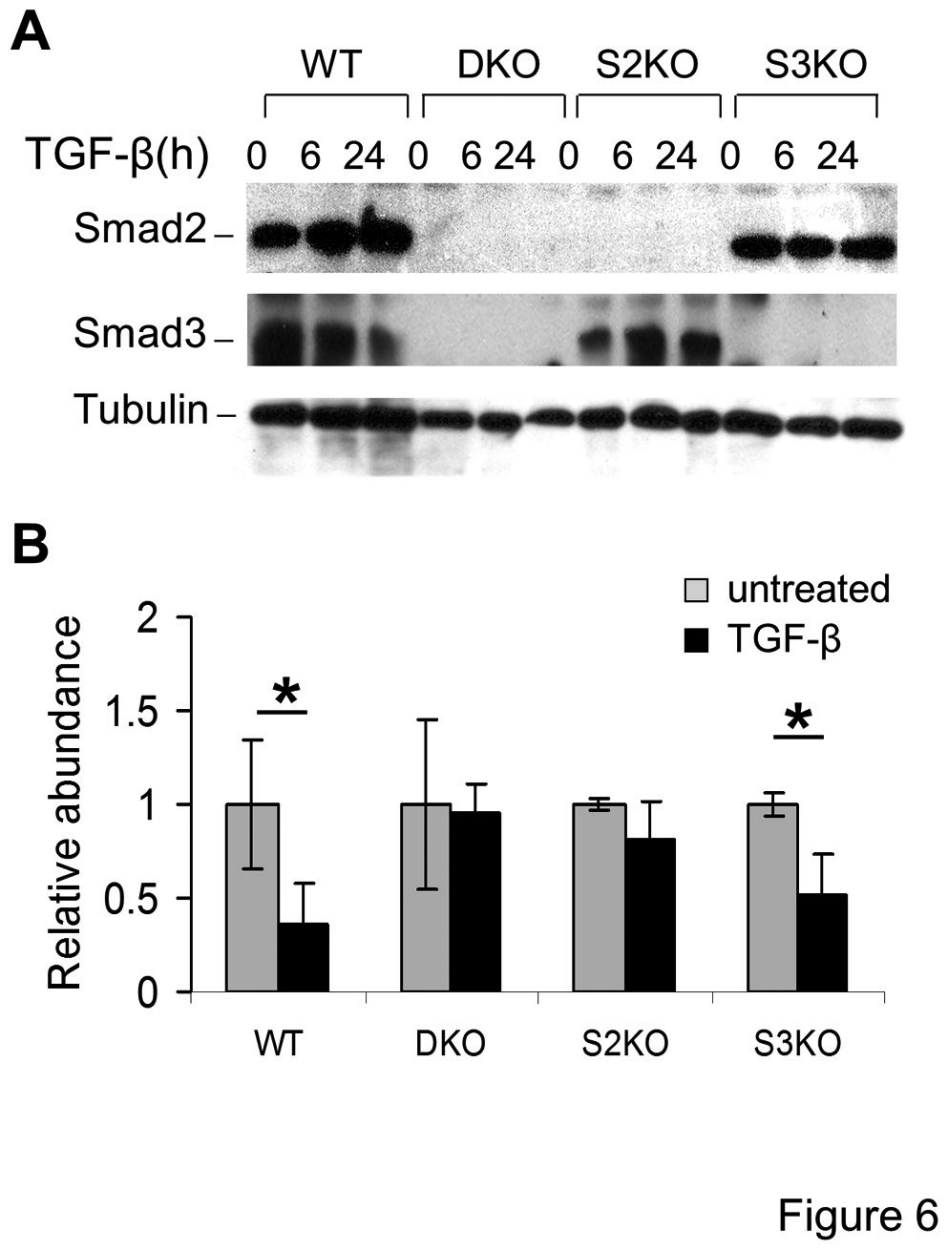
The Smad2-dependent pathway mediates the TGF-β-induced downregulation of miR-30d, and Smad3 is not required. **A**. Immunoblots depicting total Smad2 or total Smad3 protein levels in wildtype (WT), Smad2-deficient (S2KO), Smad3-deficient (S3KO), or Smad2/Smad3-double deficient (DKO) conditionally immortalized murine podocytes at baseline (0 hr) or treated with TGF-β (5 ng/ml) for 6 and 24 hr (tubulin is shown as loading control). **B**. Bar graph showing the mean ± S.D. of the relative abundance of miR-30d after 24 hr of TGF-β treatment (black bars) normalized to untreated conditions in WT, S2KO, S3KO, or DKO podocytes. * denotes p < 0.05.

We previously reported conditionally immortalized podocyte cell lines that were established from mouse strains with targeted deletion of Smad2, Smad3, or both (Smad2 + Smad3) [6]. The deficiency of Smad2, Smad3, or Smad2/Smad3 in these cell lines was confirmed by immunoblotting for total Smad2 and Smad3 in wildtype podocytes and podocytes carrying targeted deletions of Smad2 (S2KO), Smad3 (S3KO), and Smad2/Smad3 (DKO) (Figure 6A). TGF-β significantly downregulated levels of miR-30 members in wild-type podocytes and S3KO podocytes (Figure 6B). In contrast, TGF-β had no significant effect on miR-30 levels in S2KO and DKO podocytes (Figure 6B), demonstrating that Smad2 mediates the TGF-β-induced downregulation of miR-30 in podocytes.

### miR-30 downregulation is required for activation of pro-apoptotic p53 by TGF-β

**Figure 7:**
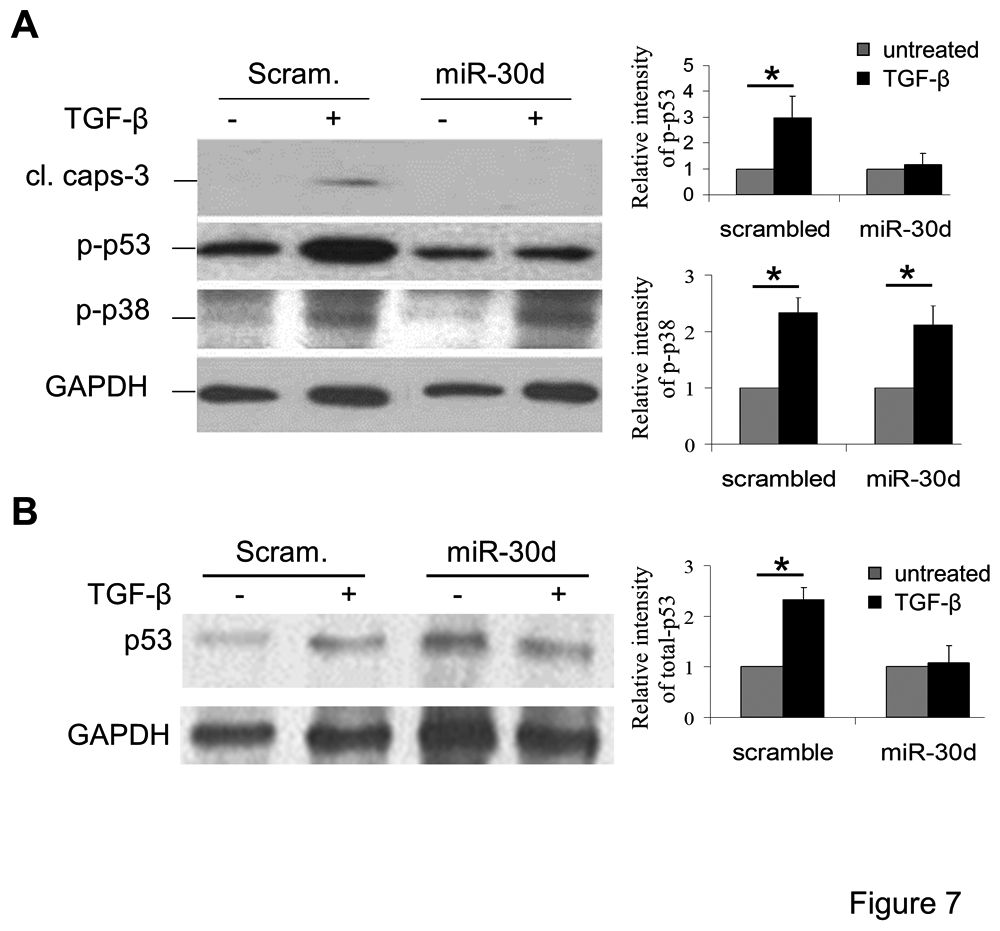
Lentiviral expression of miR-30d abrogates the TGF-β-induced expression and activation of pro-apoptotic p53. **A**. Immunoblots depict cleaved caspase-3, phosphorylated p53 (p-p53), phosphorylated p38 (p-p38), or GAPDH (loading control) in podocytes expressing scrambled miR or miR-30d left untreated (-) or treated (+) with TGF-β. **B**. Immunoblots show total p53 protein expression and GAPDH (loading control) in podocytes as described in A.

We have previously demonstrated that TGF-β-induced podocyte apoptosis requires a shift in signaling activity from anti-apoptotic PI3K/AKT to pro-apoptotic p38 MAP kinase activation [5,6,8]. TGF-β induced the phosphorylation of p38 in both control scrambled miR- or exogenous miR-30d-expressing podocytes (Figure 7A). Lentiviral miR-30d expression also did not alter the TGF-β-induced phosphorylation of p44/42 MAP kinase or Akt. In contrast, the TGF-β-induced phosphorylation of pro-apoptotic p53 observed in control podocytes was absent in lentiviral miR-30d-expressing podocytes (Figure 7A). In addition, p53 protein levels were increased by TGF-β in control cells, whereas the overexpression of miR-30d blocked the effect of TGF-β on p53 protein levels (Figure 7B). These results suggest that Smad2-dependent downregulation of miR-30 by TGF-β is required to specifically activate p53 signaling during podocyte apoptosis.

## Discussion

**Figure 8:**
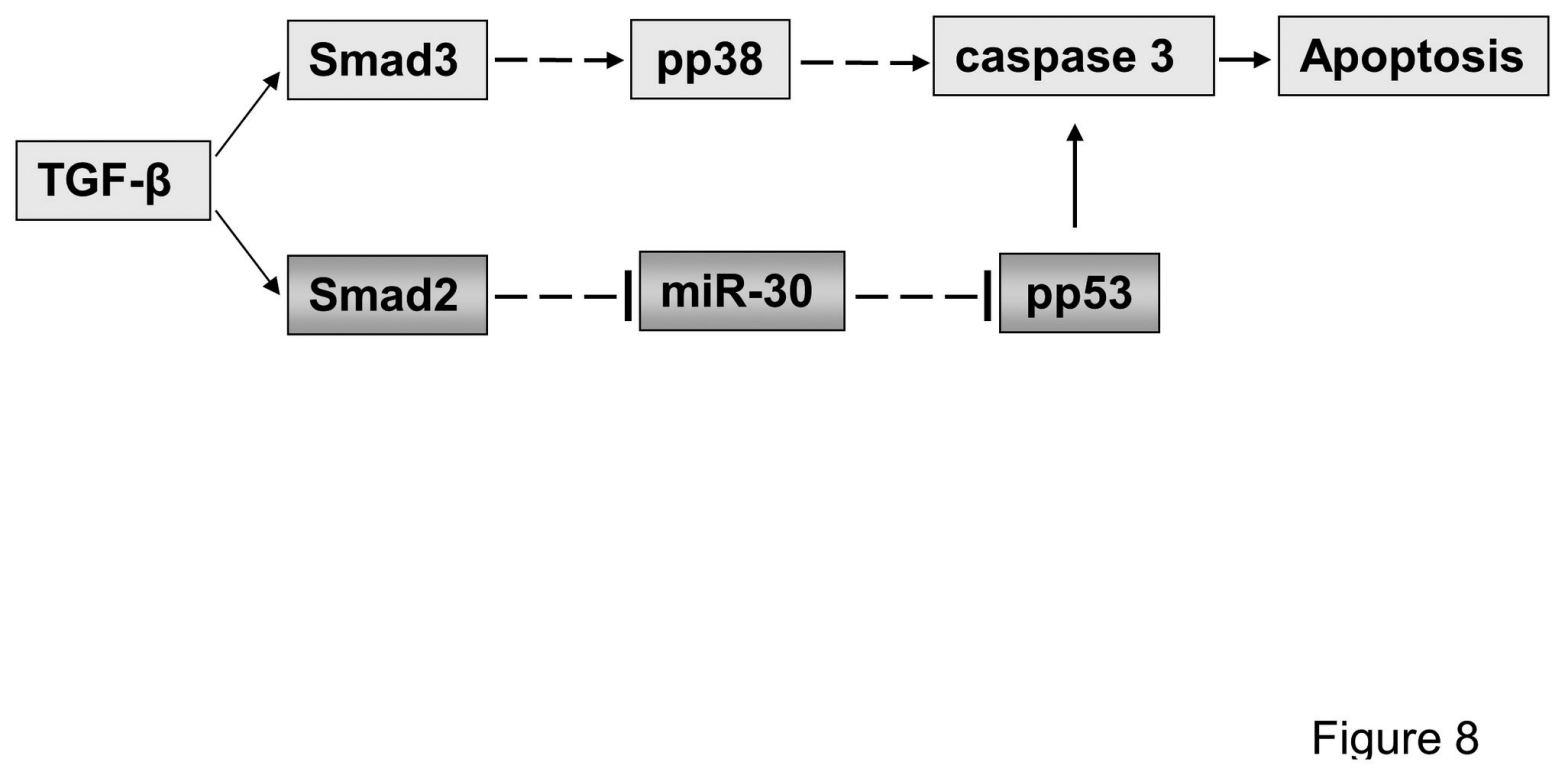
A proposed model of the distinct molecular mechanisms and functional roles of Smad2- and Smad3-dependent pathways in TGF-β-induced pro-apoptotic signaling. Smad3-dependent signaling mediates the activation of pro-apoptotic p38 required for caspase-3 activation and apoptosis [5) [8]. In contrast, Smad2-dependent signaling selectively downregulates miR-30 family transcripts to permit the activation of pro-apoptotic p53, which is required for caspase-3 activation and apoptosis.

The novel findings reported in our work connect for the first time the miR-30 family with the TGF-β/Smad signaling network. Downregulation of miR-30 members was required for TGF-β-induced apoptosis in visceral glomerular epithelial cells (podocytes). Thus, we conclude that an essential miR-30 threshold exists in podocytes, above which miR-30s can suppress pro-apoptotic factors and promote cell survival. For example, we demonstrated for the first time that to induce apoptosis in podocytes, TGF-β signaling must decrease protective miR-30 levels specifically through the Smad2-dependent pathway, whereas Smad3 is not required. Moreover, we showed that sustaining miR-30 levels above this proposed threshold prevented both increases in protein and in phosphorylation of p53 in podocytes. Thus, we propose a novel pro-apoptotic TGF-β-Smad2-miR-30-p53 pathway that is necessary for caspase-3 activation and apoptosis in podocytes (Figure 8). Together with the previously reported requirement for the activation of a pro-apoptotic TGF-β-Smad3-p38 pathway [8] and the inactivation of an anti-apoptotic TGF-β-Cd2ap-PI3K-Akt pathway [6], the current findings add novel insights into the complexity of coordinated intracellular regulatory networks that control cell survival or cell death in response to extracellular TGF-β activation in podocytes.

The finding that the TGF-β-induced downregulation of miR-30 may selectively promote apoptotic outcomes by permitting the activation of p53 expands our understanding of the emerging role of miRNAs in conferring biological specificity in cell type-dependent pluripotent TGF-β signaling networks. For example, extensive work has demonstrated that the upregulation of miR-192 [12] and miR-216a/miR-217 [14] by TGF-β in mesenchymal glomerular mesangial cells can switch off E-Box transcriptional repressors, resulting in increased collagen synthesis [12], and turn on Akt by repressing its inhibitor, PTEN, promoting mesangial cell survival and hypertrophy [14]. Increased miR-206 and miR-29 repress the translation of HDAC4, which is required in TGF-β-induced myogenic differentiation [32]. TGF-β controls the biogenesis of mature miR-21 by permitting the interaction of the pre-miR processing enzyme, Drosha, with pre-miR-21-bound Smad2 or Smad3 [33]. Mature miR-21 suppresses PDCD4, an inhibitor of contractile genes in vascular smooth muscle cells, resulting in the contractile VSMC phenotype [33]. The important role of TGF-β in controlling epithelial plasticity by promoting epithelial-to-mesenchymal transition (EMT) is well-documented [34] and is dependent on the coordinated upregulation of miR-155 and the subsequent inhibition of its target, RhoA, and the downregulation of miR-30 in mouse mammary epithelial cells [35]. Interestingly, miR-155 modulates the TGF-β-controlled expression of pro-inflammatory cytokines in macrophages by the translational repression of Smad2 via direct binding to its 3’ UTR [36]. Thus, TGF-β signaling networks predominantly upregulate distinct sets of miRs to repress key mediators controlling epithelial plasticity, myogenic differentiation, contractile properties of VSMCs, or survival and collagen synthesis of mesangial cells.

In contrast with the commonly observed miR upregulation by TGF-β, reports of TGF-β-mediated miR downregulation are rare. Concerted downregulation of all five members of the miR-200 family by TGF-β in human breast cancer cells derepressed the miR-200 target genes, ZEB1 and P 1, mediating repression of E-cadherin EMT and tumor cell invasiveness [37,38]. Similarly, our results demonstrated for the first time that the concerted downregulation of all miR-30 members was specifically required for the activation of a central mediator of apoptosis, p53, by TGF-β. Interestingly, miR-30 has recently been shown to target p53 directly in human cardiomyocytes, resulting in inhibition of Drp1-mediated mitochondrial fission and apoptosis in response to oxidative stress [20]. However, the reported inhibitory mechanism of a direct miR-30-p53 target pairing differs from that observed in our results. In our study, miR-30s controlled the functional regulation of p53 by preventing its phosphorylation by TGF-β. Thus, because the miR-30-p53 target pairing is not evolutionarily conserved and is only observed in primate genomes, our findings provide an important, previously unknown alternative mechanism for the inhibition of p53-mediated apoptosis by miR-30, at least in glomerular podocytes.

Anti-apoptotic effect of miR-30s in podocytes is in contrast with their pro-apoptotic effect that has been found in the breast tumor-initiating cells, BT-ICs [21]. Overexpression of miR-30 induced, while miR-30 reduction inhibited, the apoptosis of BT-ICs cells through affecting target Itgb3 expression. However, Itgb3 is not expressed in podocytes (data not shown), precluding the involvement of miR-30-Itgb3 pair in podocyte apoptosis. Apparently, the exact role of miR-30s in apoptosis is cell-type dependent.

Ongoing clinical trials are examining the efficacy and feasibility of several systemic inhibitors of TGF-β, including ligand-neutralizing antibodies and inhibitors of TGF-β receptor type I kinase, to prevent the progression of kidney disease, including diabetic nephropathy. However, the clinical utility of non-selective, long-term TGF-β inhibition may be limited by the risk of interfering with multiple TGF-β activities that are required for cell and tissue homeostasis, such as cell growth arrest, immunosuppression, and differentiation. Targeting selective TGF-β activities, including apoptosis, has not been possible. Because we demonstrated that miR-30 was specifically controlled by Smad2, but not Smad3, therapeutic supplementation of miR-30 may provide an approach to target pro-apoptotic TGF-β activity without interfering with homeostatic Smad3- or Cd2ap-dependent activities. Ongoing and future work will be needed to elucidate at the molecular level the mechanisms that mediate the concerted downregulation of all five miR-30 family members downstream of Smad2 and to determine how miR-30s inhibit the phosphorylation/activation of pro-apoptotic p53.

According to the prevailing ‘podocyte depletion’ paradigm of glomerular diseases, podocytes are terminally differentiated cells and are typically not replaced by efficient cell proliferation in chronic progressive glomerular disease [3,4]. Thus, the prevention of podocyte loss caused by apoptosis or other mechanisms is an important yet unrealized therapeutic goal to prevent the progression of glomerular disease. We propose that the miR-30 family represents an attractive novel therapeutic target for the protection of podocytes in glomerular diseases, as our study demonstrated that maintenance of miR-30 levels above critical thresholds prevented podocyte apoptosis in the presence of TGF-β. Indeed, therapeutic maintenance of miR-30 may protect epithelial cells, including podocytes, from multiple pro-apoptotic stressors, including TGF-β (this work) and oxidative stress and hypoxia [20]. Thus, it will be interesting to examine whether restoration of homeostatic miR-30 levels by therapeutic miR-30 replacement therapy will protect the survival of podocytes exposed to a range of common mediators of glomerular injury, including metabolic, mechanic, and toxic stressors. Rapidly increasing evidence suggests a considerable clinical potential for miR replacement therapies [39].

## Supporting Information

Figure S1 miR-30s were downregulated in the glomeruli of Alb-TGF-β transgenic mice Quantitative PCR analyses of miR-30a, - 30c and -30d were performed with the glomerular RNA samples from two-week old Alb-TGF-β mice (n = 5) and the age-matched controls (n = 4) using the method of magnetic bead perfusion. The bar graph shows the mean ± S.D. of the relative abundances of miR-30a, -30c, and -30d in the glomeruli of control and Alb-TGF-β groups. Significant difference (p < 0.05) between controls and Alb-TGF-β mice is present for all these miR-30s. Note that at the age of 2 weeks the Alb-TGF-β mice had a ~ 20% podocyte loss according to our previous studies [8], which contributed to the miR-30 reduction in the glomeruli of Alb-TGF-β mice.
(TIF)

Figure S2 miR-30 precursors were downregulated in the glomeruli of Alb-TGF-β transgenic mice The same total RNA samples in Figure S1 were used for qPCR analyses of the precursors of miR-30a -30c, and -30d following the method we described previously [16]. The Bar graph shows the mean ± S.D. of the relative abundance of the precursor of miR-30a, -30c, or -30d in the glomeruli of Alb-TGF-β mice (n = 5) and the controls (n = 4). * p < 0.05; ** p < 0.01.
(TIF)

File S1 Supporting Tables Table S1, 155 miR-30 targets that are commonly predicted by TargetScan, PicTar, and miRbase, and conserved among human, dog, mouse and rat. Table S2, List of cell death associated genes from the 155 predicted miR-30 targets according to analyses of Inguinity System.
(DOC)
