# Supplementary figures and images for: Smad2-Dependent Downregulation of miR-30 Is Required for TGF-β-Induced Apoptosis in Podocytes
Source: PLoS One. 2013 Sep 26;8(9):e75572. doi: 10.1371/journal.pone.0075572 (PMC3784460; doi:10.1371/journal.pone.0075572)

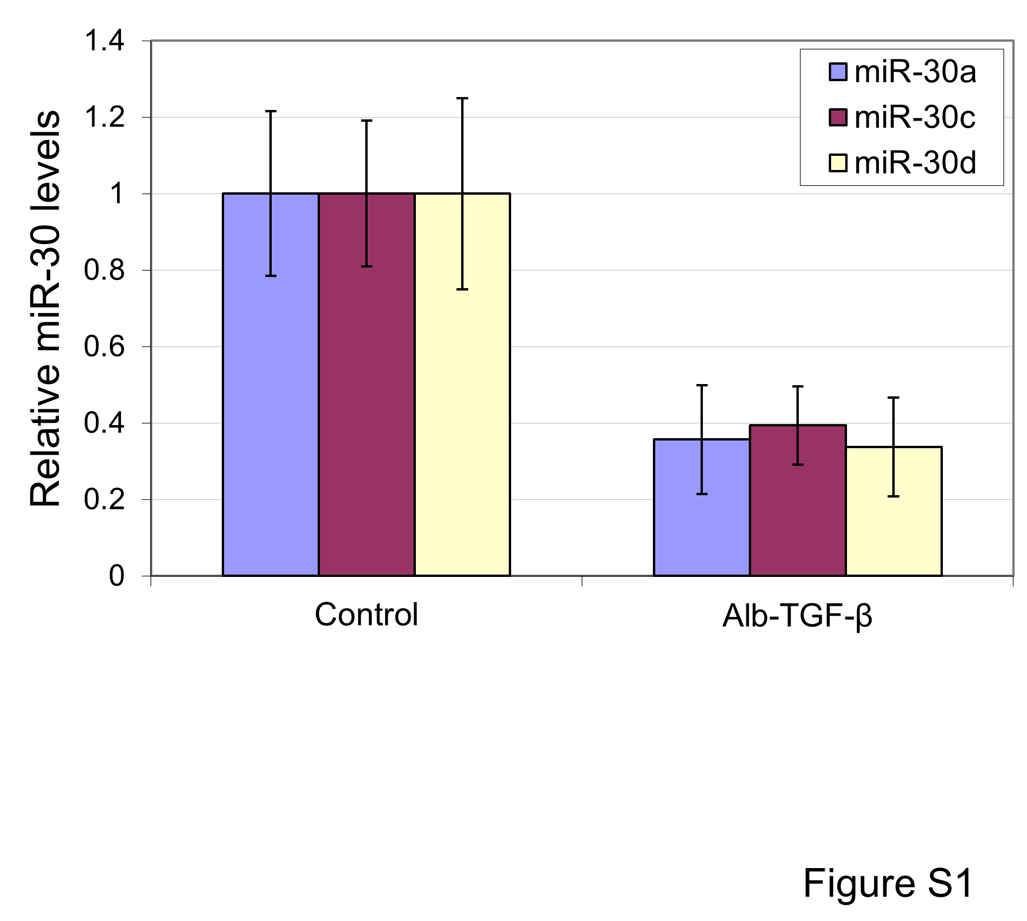

Supplement: Figure S1 — Quantitative PCR analyses of miR-30a, - 30c and -30d were performed with the glomerular RNA samples from two-week old Alb-TGF-β mice (n = 5) and the age-matched controls (n = 4) using the method of magnetic bead perfusion. The bar graph shows the mean ± S.D. of the relative abundances of miR-30a, -30c, and -30d in the glomeruli of control and Alb-TGF-β groups. Significant difference (p < 0.05) between controls and Alb-TGF-β mice is present for all these miR-30s. Note that at the age of 2 weeks the Alb-TGF-β mice had a ~ 20% podocyte loss according to our previous studies [8], which contributed to the miR-30 reduction in the glomeruli of Alb-TGF-β mice. (TIF) [file pone.0075572.s001.tif]

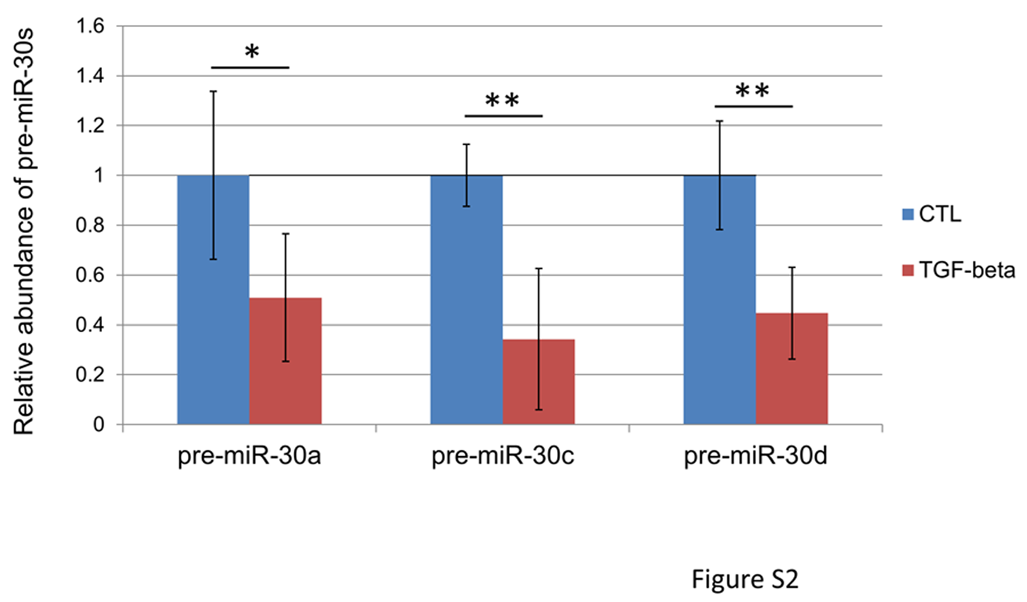

Supplement: Figure S2 — The same total RNA samples in Figure S1 were used for qPCR analyses of the precursors of miR-30a -30c, and -30d following the method we described previously [16]. The Bar graph shows the mean ± S.D. of the relative abundance of the precursor of miR-30a, -30c, or -30d in the glomeruli of Alb-TGF-β mice (n = 5) and the controls (n = 4). * p < 0.05; ** p < 0.01. (TIF) [file pone.0075572.s002.tif]
