# Supplementary material for: Smad2-Dependent Downregulation of miR-30 Is Required for TGF-β-Induced Apoptosis in Podocytes
Source: PLoS One. 2013 Sep 26;8(9):e75572. doi: 10.1371/journal.pone.0075572 (PMC3784460; doi:10.1371/journal.pone.0075572)
Supplement: File S1 — Table S1, 155 miR-30 targets that are commonly predicted by TargetScan, PicTar, and miRbase, and conserved among human, dog, mouse and rat. Table S2, List of cell death associated genes from the 155 predicted miR-30 targets according to analyses of Inguinity System. (DOC) [file pone.0075572.s003.doc]

Supporting informatio

| **Gene Symbol**  **Table S1.** 155 miR-30 targets that are commonly predicted by TargetScan, PicTar, and miRbase, and conserved among human, dog, mouse and rat. | **Gene Name** |
| --- | --- |
| ACVR1 | [activin a receptor, type i](http://david.abcc.ncifcrf.gov/geneReportFull.jsp?rowids=2866683) |
| ADRB1 | [adrenergic, beta-1-, receptor](http://david.abcc.ncifcrf.gov/geneReportFull.jsp?rowids=2870601) |
| ANKHD1 | [ankyrin repeat and kh domain containing 1](http://david.abcc.ncifcrf.gov/geneReportFull.jsp?rowids=2869596) |
| ANKRA2 | [ankyrin repeat, family a (rfxank-like), 2](http://david.abcc.ncifcrf.gov/geneReportFull.jsp?rowids=2878463) |
| AP2A1 | [adaptor-related protein complex 2, alpha 1 subunit](http://david.abcc.ncifcrf.gov/geneReportFull.jsp?rowids=2875553) |
| AP3S1 | [adaptor-related protein complex 3, sigma 1 subunit](http://david.abcc.ncifcrf.gov/geneReportFull.jsp?rowids=2872282) |
| ARID1A | [at rich interactive domain 1a (swi- like)](http://david.abcc.ncifcrf.gov/geneReportFull.jsp?rowids=3016827) |
| ARID3A | [at rich interactive domain 3a (bright- like)](http://david.abcc.ncifcrf.gov/geneReportFull.jsp?rowids=2865102) |
| ARID4A | [at rich interactive domain 4a (rbp1-like)](http://david.abcc.ncifcrf.gov/geneReportFull.jsp?rowids=2934180) |
| ASB3 | [ankyrin repeat and socs box-containing 3](http://david.abcc.ncifcrf.gov/geneReportFull.jsp?rowids=2866882) |
| ATP2A2 | [atpase, ca++ transporting, cardiac muscle, slow twitch 2](http://david.abcc.ncifcrf.gov/geneReportFull.jsp?rowids=2874195) |
| ATP2B2 | [atpase, ca++ transporting, plasma membrane 2](http://david.abcc.ncifcrf.gov/geneReportFull.jsp?rowids=2872332) |
| BAZ2B | [dkfzp434h071 protein](http://david.abcc.ncifcrf.gov/geneReportFull.jsp?rowids=2873561) |
| BCL9 | [b-cell cll/lymphoma 9](http://david.abcc.ncifcrf.gov/geneReportFull.jsp?rowids=2862993) |
| BCOR | [bcl6 co-repressor](http://david.abcc.ncifcrf.gov/geneReportFull.jsp?rowids=2864716) |
| BECN1 | [beclin 1 (coiled-coil, myosin-like bcl2 interacting protein)](http://david.abcc.ncifcrf.gov/geneReportFull.jsp?rowids=3023891) |
| C10orf56 | [chromosome 10 open reading frame 56](http://david.abcc.ncifcrf.gov/geneReportFull.jsp?rowids=2870309) |
| C13orf18 | [chromosome 13 open reading frame 18](http://david.abcc.ncifcrf.gov/geneReportFull.jsp?rowids=2867898) |
| C13orf23 | [chromosome 13 open reading frame 23](http://david.abcc.ncifcrf.gov/geneReportFull.jsp?rowids=2870608) |
| C20orf23 | [chromosome 20 open reading frame 23](http://david.abcc.ncifcrf.gov/geneReportFull.jsp?rowids=2878822) |
| C9orf86 | [chromosome 9 open reading frame 86](http://david.abcc.ncifcrf.gov/geneReportFull.jsp?rowids=3027804) |
| CACNB2 | [calcium channel, voltage-dependent, beta 2 subunit](http://david.abcc.ncifcrf.gov/geneReportFull.jsp?rowids=2860576) |
| CADPS | [ca2+-dependent secretion activator](http://david.abcc.ncifcrf.gov/geneReportFull.jsp?rowids=3026943) |
| CALCR | [calcitonin receptor](http://david.abcc.ncifcrf.gov/geneReportFull.jsp?rowids=2872499) |
| CCNK | [cyclin k](http://david.abcc.ncifcrf.gov/geneReportFull.jsp?rowids=2869910) |
| CHD9 | [hypothetical protein bc022889](http://david.abcc.ncifcrf.gov/geneReportFull.jsp?rowids=2859003) |
| CHST1 | [carbohydrate (keratan sulfate gal-6) sulfotransferase 1](http://david.abcc.ncifcrf.gov/geneReportFull.jsp?rowids=2873883) |
| CHST2 | [carbohydrate (n-acetylglucosamine-6-o) sulfotransferase 2](http://david.abcc.ncifcrf.gov/geneReportFull.jsp?rowids=2873470) |
| COL13A1 | [collagen, type xiii, alpha 1](http://david.abcc.ncifcrf.gov/geneReportFull.jsp?rowids=2878759) |
| COPS7B | [cop9 constitutive photomorphogenic homolog subunit 7b (arabidopsis)](http://david.abcc.ncifcrf.gov/geneReportFull.jsp?rowids=3026952) |
| CPSF6 | [cleavage and polyadenylation specific factor 6, 68kda](http://david.abcc.ncifcrf.gov/geneReportFull.jsp?rowids=2873577) |
| CSDA | [cold shock domain protein a](http://david.abcc.ncifcrf.gov/geneReportFull.jsp?rowids=2866477) |
| CUL2 | [cullin 2](http://david.abcc.ncifcrf.gov/geneReportFull.jsp?rowids=3027704) |
| DCX | [doublecortex; lissencephaly, x-linked (doublecortin)](http://david.abcc.ncifcrf.gov/geneReportFull.jsp?rowids=2866165) |
| DDIT4 | [dna-damage-inducible transcript 4](http://david.abcc.ncifcrf.gov/geneReportFull.jsp?rowids=2860813) |
| DET1 | [de-etiolated homolog 1 (arabidopsis)](http://david.abcc.ncifcrf.gov/geneReportFull.jsp?rowids=3029789) |
| DGKZ | [diacylglycerol kinase, zeta 104kda](http://david.abcc.ncifcrf.gov/geneReportFull.jsp?rowids=2863959) |
| DLG5 | [discs, large homolog 5 (drosophila)](http://david.abcc.ncifcrf.gov/geneReportFull.jsp?rowids=2875071) |
| DLGAP4 | [discs, large (drosophila) homolog-associated protein 4](http://david.abcc.ncifcrf.gov/geneReportFull.jsp?rowids=3025845) |
| DLL4 | [delta-like 4 (drosophila)](http://david.abcc.ncifcrf.gov/geneReportFull.jsp?rowids=2862465) |
| DOC2A | [double c2-like domains, alpha](http://david.abcc.ncifcrf.gov/geneReportFull.jsp?rowids=2874385) |
| DOCK7 | [dedicator of cytokinesis 7](http://david.abcc.ncifcrf.gov/geneReportFull.jsp?rowids=2876712) |
| DPYSL2 | [dihydropyrimidinase-like 2](http://david.abcc.ncifcrf.gov/geneReportFull.jsp?rowids=3027993) |
| EED | [embryonic ectoderm development](http://david.abcc.ncifcrf.gov/geneReportFull.jsp?rowids=3028510) |
| EPC2 | [enhancer of polycomb homolog 2 (drosophila)](http://david.abcc.ncifcrf.gov/geneReportFull.jsp?rowids=2881460) |
| EPHB2 | [eph receptor b2](http://david.abcc.ncifcrf.gov/geneReportFull.jsp?rowids=2865147) |
| FAM40A | [family with sequence similarity 40, member a](http://david.abcc.ncifcrf.gov/geneReportFull.jsp?rowids=2865291) |
| FAM43A | [family with sequence similarity 43, member a](http://david.abcc.ncifcrf.gov/geneReportFull.jsp?rowids=2869882) |
| FAP | [fibroblast activation protein, alpha](http://david.abcc.ncifcrf.gov/geneReportFull.jsp?rowids=3028650) |
| FBXL20 | [f-box and leucine-rich repeat protein 20](http://david.abcc.ncifcrf.gov/geneReportFull.jsp?rowids=2859053) |
| FKBP3 | [fk506 binding protein 3, 25kda](http://david.abcc.ncifcrf.gov/geneReportFull.jsp?rowids=2860310) |
| FOXG1B | [forkhead box g1b](http://david.abcc.ncifcrf.gov/geneReportFull.jsp?rowids=3023699) |
| FRMPD1 | [ferm and pdz domain containing 1](http://david.abcc.ncifcrf.gov/geneReportFull.jsp?rowids=3029094) |
| GALNT3 | [udp-n-acetyl-alpha-d-galactosamine:polypeptide n-acetylgalactosaminyltransferase 3 (galnac-t3)](http://david.abcc.ncifcrf.gov/geneReportFull.jsp?rowids=2865778) |
| GLDC | [glycine dehydrogenase (decarboxylating; glycine decarboxylase, glycine cleavage system protein p)](http://david.abcc.ncifcrf.gov/geneReportFull.jsp?rowids=2866588) |
| GNAI2 | [guanine nucleotide binding protein (g protein), alpha inhibiting activity polypeptide 2](http://david.abcc.ncifcrf.gov/geneReportFull.jsp?rowids=2877686) |
| GNAO1 | [guanine nucleotide binding protein (g protein), alpha activating activity polypeptide o](http://david.abcc.ncifcrf.gov/geneReportFull.jsp?rowids=2858474) |
| GRK5 | [g protein-coupled receptor kinase 5](http://david.abcc.ncifcrf.gov/geneReportFull.jsp?rowids=2869291) |
| GRM3 | [glutamate receptor, metabotropic 3](http://david.abcc.ncifcrf.gov/geneReportFull.jsp?rowids=3026450) |
| GRM5 | [glutamate receptor, metabotropic 5](http://david.abcc.ncifcrf.gov/geneReportFull.jsp?rowids=2858052) |
| HERC2 | [hect domain and rld 2](http://david.abcc.ncifcrf.gov/geneReportFull.jsp?rowids=2865506) |
| IER2 | [immediate early response 2](http://david.abcc.ncifcrf.gov/geneReportFull.jsp?rowids=2864323) |
| IHPK3 | [inositol hexaphosphate kinase 3](http://david.abcc.ncifcrf.gov/geneReportFull.jsp?rowids=2857979) |
| IL1A | [interleukin 1, alpha](http://david.abcc.ncifcrf.gov/geneReportFull.jsp?rowids=3025023) |
| IRS1 | [insulin receptor substrate 1](http://david.abcc.ncifcrf.gov/geneReportFull.jsp?rowids=3026835) |
| JAG2 | [jagged 2](http://david.abcc.ncifcrf.gov/geneReportFull.jsp?rowids=3027121) |
| JMJD1A | [jumonji domain containing 1a](http://david.abcc.ncifcrf.gov/geneReportFull.jsp?rowids=3029993) |
| KCNJ3 | [potassium inwardly-rectifying channel, subfamily j, member 3](http://david.abcc.ncifcrf.gov/geneReportFull.jsp?rowids=2870074) |
| KCTD3 | [potassium channel tetramerisation domain containing 3](http://david.abcc.ncifcrf.gov/geneReportFull.jsp?rowids=2874781) |
| KCTD5 | [potassium channel tetramerisation domain containing 5](http://david.abcc.ncifcrf.gov/geneReportFull.jsp?rowids=2869992) |
| KCTD8 | [potassium channel tetramerisation domain containing 8](http://david.abcc.ncifcrf.gov/geneReportFull.jsp?rowids=2864173) |
| LGI1 | [leucine-rich, glioma inactivated 1](http://david.abcc.ncifcrf.gov/geneReportFull.jsp?rowids=2867893) |
| LHX8 | [lim homeobox 8](http://david.abcc.ncifcrf.gov/geneReportFull.jsp?rowids=2873308) |
| LRRC17 | [leucine rich repeat containing 17](http://david.abcc.ncifcrf.gov/geneReportFull.jsp?rowids=3027820) |
| MAN1B1 | [mannosidase, alpha, class 1b, member 1](http://david.abcc.ncifcrf.gov/geneReportFull.jsp?rowids=2860208) |
| MAP3K12 | [mitogen-activated protein kinase kinase kinase 12](http://david.abcc.ncifcrf.gov/geneReportFull.jsp?rowids=2870627) |
| MAP3K5 | [mitogen-activated protein kinase kinase kinase 5](http://david.abcc.ncifcrf.gov/geneReportFull.jsp?rowids=2865683) |
| MKRN3 | [makorin, ring finger protein, 3](http://david.abcc.ncifcrf.gov/geneReportFull.jsp?rowids=2874596) |
| MMD | [monocyte to macrophage differentiation-associated](http://david.abcc.ncifcrf.gov/geneReportFull.jsp?rowids=2872072) |
| MYBL2 | [v-myb myeloblastosis viral oncogene homolog (avian)-like 2](http://david.abcc.ncifcrf.gov/geneReportFull.jsp?rowids=2871349) |
| NAGPA | [n-acetylglucosamine-1-phosphodiester alpha-n-acetylglucosaminidase](http://david.abcc.ncifcrf.gov/geneReportFull.jsp?rowids=2862843) |
| NCOR2 | [nuclear receptor co-repressor 2](http://david.abcc.ncifcrf.gov/geneReportFull.jsp?rowids=2864335) |
| NEK4 | [nima (never in mitosis gene a)-related kinase 4](http://david.abcc.ncifcrf.gov/geneReportFull.jsp?rowids=3028879) |
| NEUROD1 | [neurogenic differentiation 1](http://david.abcc.ncifcrf.gov/geneReportFull.jsp?rowids=3025295) |
| NEUROD6 | [neurogenic differentiation 6](http://david.abcc.ncifcrf.gov/geneReportFull.jsp?rowids=2878794) |
| NFIB | [nuclear factor i/b](http://david.abcc.ncifcrf.gov/geneReportFull.jsp?rowids=3026146) |
| NHLH2 | [nescient helix loop helix 2](http://david.abcc.ncifcrf.gov/geneReportFull.jsp?rowids=2869664) |
| NKX2-2 | [nk2 transcription factor related, locus 2 (drosophila)](http://david.abcc.ncifcrf.gov/geneReportFull.jsp?rowids=2876629) |
| NR6A1 | [nuclear receptor subfamily 6, group a, member 1](http://david.abcc.ncifcrf.gov/geneReportFull.jsp?rowids=2860611) |
| OMG | [oligodendrocyte myelin glycoprotein](http://david.abcc.ncifcrf.gov/geneReportFull.jsp?rowids=3028260) |
| P4HA2 | [procollagen-proline, 2-oxoglutarate 4-dioxygenase (proline 4-hydroxylase), alpha polypeptide ii](http://david.abcc.ncifcrf.gov/geneReportFull.jsp?rowids=3016233) |
| PAPD4 | [pap associated domain containing 4](http://david.abcc.ncifcrf.gov/geneReportFull.jsp?rowids=2860456) |
| PAWR | [prkc, apoptosis, wt1, regulator](http://david.abcc.ncifcrf.gov/geneReportFull.jsp?rowids=2858957) |
| PAX3 | [paired box gene 3 (waardenburg syndrome 1)](http://david.abcc.ncifcrf.gov/geneReportFull.jsp?rowids=3025006) |
| PCDH10 | [protocadherin 10](http://david.abcc.ncifcrf.gov/geneReportFull.jsp?rowids=2874171) |
| PGGT1B | [protein geranylgeranyltransferase type i, beta subunit](http://david.abcc.ncifcrf.gov/geneReportFull.jsp?rowids=2926682) |
| PGM1 | [phosphoglucomutase 1](http://david.abcc.ncifcrf.gov/geneReportFull.jsp?rowids=2865613) |
| PHTF2 | [putative homeodomain transcription factor 2](http://david.abcc.ncifcrf.gov/geneReportFull.jsp?rowids=2860539) |
| PIK3R2 | [phosphoinositide-3-kinase, regulatory subunit 2 (p85 beta)](http://david.abcc.ncifcrf.gov/geneReportFull.jsp?rowids=2876889) |
| PLCB4 | [phospholipase c, beta 4](http://david.abcc.ncifcrf.gov/geneReportFull.jsp?rowids=2870620) |
| PNN | [pinin, desmosome associated protein](http://david.abcc.ncifcrf.gov/geneReportFull.jsp?rowids=2872383) |
| PPARGC1B | [peroxisome proliferative activated receptor, gamma, coactivator 1, beta](http://david.abcc.ncifcrf.gov/geneReportFull.jsp?rowids=2873860) |
| PRICKLE1 | [prickle-like 1 (drosophila)](http://david.abcc.ncifcrf.gov/geneReportFull.jsp?rowids=2859322) |
| PRLR | [prolactin receptor](http://david.abcc.ncifcrf.gov/geneReportFull.jsp?rowids=2874782) |
| PSMD7 | [proteasome (prosome, macropain) 26s subunit, non-atpase, 7 (mov34 homolog)](http://david.abcc.ncifcrf.gov/geneReportFull.jsp?rowids=3026022) |
| PTGFRN | [prostaglandin f2 receptor negative regulator](http://david.abcc.ncifcrf.gov/geneReportFull.jsp?rowids=3029697) |
| PTPN13 | [protein tyrosine phosphatase, non-receptor type 13 (apo-1/cd95 (fas)-associated phosphatase)](http://david.abcc.ncifcrf.gov/geneReportFull.jsp?rowids=2873117) |
| RAB32 | [rab32, member ras oncogene family](http://david.abcc.ncifcrf.gov/geneReportFull.jsp?rowids=2862717) |
| RAB38 | [rab38, member ras oncogene family](http://david.abcc.ncifcrf.gov/geneReportFull.jsp?rowids=2862830) |
| RANBP10 | [ran binding protein 10](http://david.abcc.ncifcrf.gov/geneReportFull.jsp?rowids=2873181) |
| RANBP9 | [ran binding protein 9](http://david.abcc.ncifcrf.gov/geneReportFull.jsp?rowids=3030107) |
| RASA1 | [ras p21 protein activator (gtpase activating protein) 1](http://david.abcc.ncifcrf.gov/geneReportFull.jsp?rowids=2883843) |
| RASD1 | [ras, dexamethasone-induced 1](http://david.abcc.ncifcrf.gov/geneReportFull.jsp?rowids=2865694) |
| RFXDC1 | [regulatory factor x domain containing 1](http://david.abcc.ncifcrf.gov/geneReportFull.jsp?rowids=2859615) |
| RGS2 | [regulator of g-protein signalling 2, 24kda](http://david.abcc.ncifcrf.gov/geneReportFull.jsp?rowids=2877293) |
| RHEBL1 | [ras homolog enriched in brain like 1](http://david.abcc.ncifcrf.gov/geneReportFull.jsp?rowids=2877922) |
| RHOB | [ras homolog gene family, member b](http://david.abcc.ncifcrf.gov/geneReportFull.jsp?rowids=2863728) |
| RKHD3 | [ring finger and kh domain containing 3](http://david.abcc.ncifcrf.gov/geneReportFull.jsp?rowids=2865460) |
| RNF122 | [ring finger protein 122](http://david.abcc.ncifcrf.gov/geneReportFull.jsp?rowids=2861499) |
| RRAD | [ras-related associated with diabetes](http://david.abcc.ncifcrf.gov/geneReportFull.jsp?rowids=2864063) |
| RRAS2 | [related ras viral (r-ras) oncogene homolog 2](http://david.abcc.ncifcrf.gov/geneReportFull.jsp?rowids=3025567) |
| SAP30 | [sin3a-associated protein, 30kda](http://david.abcc.ncifcrf.gov/geneReportFull.jsp?rowids=2867132) |
| SBF1 | [set binding factor 1](http://david.abcc.ncifcrf.gov/geneReportFull.jsp?rowids=2898042) |
| SCN3A | [sodium channel, voltage-gated, type iii, alpha](http://david.abcc.ncifcrf.gov/geneReportFull.jsp?rowids=2874835) |
| SCN8A | [sodium channel, voltage gated, type viii, alpha](http://david.abcc.ncifcrf.gov/geneReportFull.jsp?rowids=2875243) |
| SEC23A | [sec23 homolog a (s. cerevisiae)](http://david.abcc.ncifcrf.gov/geneReportFull.jsp?rowids=2860516) |
| SEMA3A | [sema domain, immunoglobulin domain (ig), short basic domain, secreted, (semaphorin) 3a](http://david.abcc.ncifcrf.gov/geneReportFull.jsp?rowids=2865710) |
| SEMA6D | [kiaa1479 protein](http://david.abcc.ncifcrf.gov/geneReportFull.jsp?rowids=2859169) |
| SERPINE1 | [serpin peptidase inhibitor, clade e (nexin, plasminogen activator inhibitor type 1), member 1](http://david.abcc.ncifcrf.gov/geneReportFull.jsp?rowids=2861452) |
| SLC25A14 | [solute carrier family 25 (mitochondrial carrier, brain), member 14](http://david.abcc.ncifcrf.gov/geneReportFull.jsp?rowids=2873473) |
| SLC41A2 | [solute carrier family 41, member 2](http://david.abcc.ncifcrf.gov/geneReportFull.jsp?rowids=3028161) |
| SLC7A10 | [hypothetical protein flj20839](http://david.abcc.ncifcrf.gov/geneReportFull.jsp?rowids=2873210) |
| SMARCD2 | [swi/snf related, matrix associated, actin dependent regulator of chromatin, subfamily d, member 2](http://david.abcc.ncifcrf.gov/geneReportFull.jsp?rowids=2860658) |
| SNAI1 | [snail homolog 1 (drosophila)](http://david.abcc.ncifcrf.gov/geneReportFull.jsp?rowids=2859569) |
| SNX16 | [sorting nexin 16](http://david.abcc.ncifcrf.gov/geneReportFull.jsp?rowids=3027601) |
| SOCS1 | [suppressor of cytokine signaling 1](http://david.abcc.ncifcrf.gov/geneReportFull.jsp?rowids=2874883) |
| SON | [son dna binding protein](http://david.abcc.ncifcrf.gov/geneReportFull.jsp?rowids=2864618) |
| STAG2 | [stromal antigen 2](http://david.abcc.ncifcrf.gov/geneReportFull.jsp?rowids=2875989) |
| STIM2 | [stromal interaction molecule 2](http://david.abcc.ncifcrf.gov/geneReportFull.jsp?rowids=2873003) |
| STK39 | [serine threonine kinase 39 (ste20/sps1 homolog, yeast)](http://david.abcc.ncifcrf.gov/geneReportFull.jsp?rowids=3030133) |
| SUPT3H | [suppressor of ty 3 homolog (s. cerevisiae)](http://david.abcc.ncifcrf.gov/geneReportFull.jsp?rowids=2875240) |
| SYNGR3 | [synaptogyrin 3](http://david.abcc.ncifcrf.gov/geneReportFull.jsp?rowids=3023924) |
| TIA1 | [tia1 cytotoxic granule-associated rna binding protein](http://david.abcc.ncifcrf.gov/geneReportFull.jsp?rowids=2876637) |
| TLL2 | [tolloid-like 2](http://david.abcc.ncifcrf.gov/geneReportFull.jsp?rowids=2858820) |
| TMEFF1 | [transmembrane protein with egf-like and two follistatin-like domains 1](http://david.abcc.ncifcrf.gov/geneReportFull.jsp?rowids=2864036) |
| TMEM16D | [transmembrane protein 16d](http://david.abcc.ncifcrf.gov/geneReportFull.jsp?rowids=2870782) |
| TNRC15 | [trinucleotide repeat containing 15](http://david.abcc.ncifcrf.gov/geneReportFull.jsp?rowids=2862639) |
| TNXB | [tenascin xb](http://david.abcc.ncifcrf.gov/geneReportFull.jsp?rowids=2876292) |
| UBE2I | [ubiquitin-conjugating enzyme e2i (ubc9 homolog, yeast)](http://david.abcc.ncifcrf.gov/geneReportFull.jsp?rowids=2860673) |
| UNC5C | [unc-5 homolog c (c. elegans)](http://david.abcc.ncifcrf.gov/geneReportFull.jsp?rowids=2871093) |
| USP44 | [ubiquitin specific peptidase 44](http://david.abcc.ncifcrf.gov/geneReportFull.jsp?rowids=2868596) |
| USP48 | [hypothetical protein flj11328](http://david.abcc.ncifcrf.gov/geneReportFull.jsp?rowids=3028759) |
| ZFYVE26 | [zinc finger, fyve domain containing 26](http://david.abcc.ncifcrf.gov/geneReportFull.jsp?rowids=2872949) |
| ZNF644 | [hypothetical protein bm-005](http://david.abcc.ncifcrf.gov/geneReportFull.jsp?rowids=2876246) |
| ZNRF1 | [hypothetical protein dkfzp434e229](http://david.abcc.ncifcrf.gov/geneReportFull.jsp?rowids=3023955) |

**Table S2.** List of cell death associated genes from the 155 predicted miR-30 targets according to analyses of Inguinity System

| **© 2000-2008 Ingenuity Systems, Inc.** All rights reserved. | | |  |  |  |
| --- | --- | --- | --- | --- | --- |
| Category | Function | Function Annotation | P-value | Genes | No. |
| Cell Death | cell death | cell death | 2.06E-02 | ACVR1, ADRB1, ATP2A2, BECN1, CACNB2, CALCR, CUL2, DDIT4, FAP, GNAO1, GRM3, GRM5, IL1A, IRS1, JAG2, MAP3K5, MAP3K12, MYBL2, NCOR2, NEUROD1, PAWR, PAX3, PIK3R2, PRLR, PTPN13, RASA1, RASD1, RHOB, RRAS2, SEMA3A, SERPINE1, SOCS1, TIA1, UNC5C | 34 |
| Cell Death | apoptosis | apoptosis | 2.25E-02 | ACVR1, ADRB1, CALCR, CUL2, DDIT4, FAP, GNAO1, GRM5, IL1A, IRS1, JAG2, MAP3K5, MAP3K12, MYBL2, NCOR2, NEUROD1, PAWR, PAX3, PIK3R2, PRLR, PTPN13 , RASA1, RASD1, RHOB, RRAS2, SEMA3A, SERPINE1, SOCS1, TIA1, UNC5C | 30 |
